# Target dependent femtosecond laser plasma implantation dynamics in enabling silica for high density erbium doping

**DOI:** 10.1038/srep14037

**Published:** 2015-09-15

**Authors:** Jayakrishnan Chandrappan, Matthew Murray, Tarun Kakkar, Peter Petrik, Emil Agocs, Zsolt Zolnai, D.P. Steenson, Animesh Jha, Gin Jose

**Affiliations:** 1 Institute for Materials Research, School of Chemical and Process Engineering, Faculty of Engineering, University of Leeds, LS2 9 JT, UK; 2 Centre for Energy Research, Institute of Technical Physics and Materials Science (MFA), H-1121 Budapest, Konkoly-Thege M. út 29-33, Hungary; 3 Institute of Microwave and photonics, Electronic and Electrical Engineering, Faculty of Engineering, University of Leeds, LS2 9JT, UK

## Abstract

Chemical dissimilarity of tellurium oxide with silica glass increases phase separation and crystallization tendency when mixed and melted for making a glass. We report a novel technique for incorporating an Er^3+^-doped tellurite glass composition into silica substrates through a femtosecond (fs) laser generated plasma assisted process. The engineered material consequently exhibits the spectroscopic properties of Er^3+^-ions, which are unachievable in pure silica and implies this as an ideal material for integrated photonics platforms. Formation of a well-defined metastable and homogeneous glass structure with Er^3+^-ions in a silica network, modified with tellurite has been characterized using high-resolution cross-sectional transmission electron microscopy (HRTEM). The chemical and structural analyses using HRTEM, Rutherford backscattering spectrometry (RBS) and laser excitation techniques, confirm that such fs-laser plasma implanted glasses may be engineered for significantly higher concentration of Er^3+^-ions without clustering, validated by the record high lifetime-density product 0.96 × 10^19^ s.cm^−3^. Characterization of planar optical layers and photoluminescence emission spectra were undertaken to determine their thickness, refractive indices and photoluminescence properties, as a function of Er^3+^ concentration via different target glasses. The increased Er^3+^ content in the target glass enhance the refractive index and photoluminescence intensity of the modified silica layer whilst the lifetime and thickness decrease.

The technological advances in optoelectronics demand a higher level of integration for multifunctional devices in a compatible optical platform than it is currently possible with fibres and planar lightwave circuits. Furthermore, photonic sensors for analytical and diagnostic applications invite the precise engineering of optical materials for well-defined waveguide structures, demonstrating high-quality mode fields for device applications. The growing demand for Tbps/cm^2^ transmission capacity necessitated the improvement of functional optical materials with suitable dopants for dense optical interconnects and its hierarchical integration with semiconductor devices^1^. Silica glasses doped with rare-earth elements are one such material, which has been actively explored for the development of miniature optical waveguide amplifiers and thin disk lasers^2^,^3^. Silica as an optical layer may be easily integrated with silicon photonic systems^4^,^5^, which are being driven for the integration of state-of-the-art CMOS devices with photonic circuitries for the next generation system in package (SiP) solutions^6^. Designing such complex and compact photonic architecture introduces inherent loss, and the provision of on-chip gain for signal amplification is an enabling capability. This is apparent in active-passive integration, especially array waveguides and power splitters, such as found in dense wavelength division multiplexing (DWDM) circuits. The distributed losses in many passive optical structures degrade signal-to-noise ratios (SNRs) and increases Bit-error-rates (BERs), so high specific gain waveguide amplifiers are very much desirable. Since the rare-earth ions cannot be dissolved into silica glass^7^, in the required concentrations, the dielectric based integrated planar amplifiers are impossible to realize at present.

In this context, an optical platform that contains erbium (Er) concentrations as high as 10^21^–10^22^ cm^−3^ and longer photoluminescence (PL) lifetime of the order of milliseconds is of great interest^8^. This will help the miniaturization of optical amplifiers and benefit the loss compensation for the integrated optic circuits. The current fabrication methods clearly exhibit concentration quenching effect even at a low Er density of 10^20^ cm^−3^ in silica. This prevents the progress of a highly doped rare earth silicate and hence it is worth exploring innovative ideas to improve the solubility of rare earth elements in silica for realizing planar amplifiers and loss-less waveguides.

Unlike silica, tellurite based glasses offer structural sites for incorporating the rare-earth ions, resulting in optical gains of 2 dB/cm^9^,^10^,^11^. On the other hand, tellurites could not support the practical applications as they are chemically very unstable in the absence of stabilizing oxides. The integration of rare-earth doped tellurite glass into silica could offer a potential solution for the current doping limitations of silica and their combination in oxygen rich network might produce a durable glassy system. From this perspective, we recently reported a femtosecond (fs) pulsed laser-assisted doping of multiple ions in silica and silicon^12^,^13^,^14^. fs lasers are widely accepted as a reliable tool for advanced material processing, micromachining and nano-ablation of a number of materials including transparent substances^15^,^16^. Our fs laser-assisted doping process involves the ablation of materials from an Er^3+^-doped tellurite glass target, using high energy fs-laser pulses that generates a plasma of energetic ions which are then implanted into a heated silica substrate at 700 °C. More specifically, the focused laser beam is absorbed by the target surface and consequently generates highly energetic plasma. The plasma plume expands away from the target at several km/s; materials within the plasma accelerate towards the substrate and hit the surface, allowing near-stoichiometric transfer of the target material into the substrate. The interfacial reaction between tellurite plasma and silica may also be assisted by thermal diffusion^17^, resulting in the formation of a homogeneous Er doped tellurite modified silica (EDTS) layer suitable for optical applications at 1550 nm wavelength.

In the present study, we investigated the process of fs-pulsed laser ablation of Er^3+^-doped zinc sodium tellurite glass and its implantation into silica substrates. The Er^3+^-ion concentrations in the target glass were varied for analysing the dynamics of EDTS layer formation and the related spectroscopic properties, so that the modified material may be optimized for optical waveguide engineering.

## Experimental

Er^3+^-doped-tellurite target glasses were prepared with a typical molar composition of (80-x)TeO_2_-10ZnO-10Na_2_O-xEr_2_O_3_, where x = 0.125, 0.25, 0.5, 0.75, 1 and 1.25 mol%. A standard batch melting and quenching process was used for the glass preparation^18^. The target glasses were prepared in 15 g batch weights using analytical grade chemicals having a purity >99.99%. After melting, the glass was cast into a brass mould and then annealed. From these cast samples, the targets were prepared by fine polishing to achieve a smooth surface. This is necessary for maintaining uniform ablation during laser irradiation. The polished glass was then fixed on to a target holder inside the process chamber. The silica glass substrate (with a size of 30 (L) × 20 (W) × 1(H) mm) was positioned 70 mm above the target and kept at a temperature of 750 °C. A Ti-sapphire femtosecond pulsed laser, operating at 800 nm wavelength with 100 fs pulse width, was focused at an angle of 60° onto the target surface. The process chamber was evacuated to a base vacuum level of 10^−6^ Torr after which oxygen was introduced to maintain an operating pressure of 80 mTorr, which has proven essential for preventing the loss of oxygen from the target surface. The target glass ablation was carried out with a laser pulse energy at 50 μJ and a repetition rate of 1 kHz. The spot size was 27 μm with an estimated peak intensity of 8.61 × 10^13^ W/cm^2^. Each test run lasted for 4 hours. Both the target and substrate were rotated at a speed of 40 rpm and 20 rpm, respectively. Moreover, the rastering of the laser beam on the target surface was enabled by a variable raster movement of the target with respect to the fixed laser beam. Target rastering and substrate rotations are essential for achieving a uniform ablation, which affects the quality of implanted surface. Post process substrate cooling was managed by ramping down the temperature at a rate of 10 °C/minute.

Structural characterization was carried out using a high resolution cross-sectional transmission electron microscope (TEM - FEI Tecnai TF20 FEG, at 200 kV) fitted with a high angle annular dark field (HAADF) detector; a Gatan SC600 Orius CCD camera (Gatan Inc., Pleasanton, CA); and an Oxford Instruments 80 mm^2^ X-max SDD energy dispersive X-ray spectroscopy (EDX) detector (Oxford Instruments plc., Abingdon, UK). The Rutherford backscattering spectrometry (RBS) analysis was performed in a scattering chamber with a two-axis goniometer connected to a 5 MV Van de Graaff accelerator at the Wigner RMI Institute of the Hungarian Academy of Sciences. The 2820 keV ^4^He^+^-ion analysing beam was collimated with two sets of four-sector slits to the spot size of 0.5 × 0.5 mm^2^, while the beam divergence was kept below 0.06°. The beam current was measured using a transmission Faraday cup. The energy resolution of the detection system was 20 keV. The spectra were recorded for sample tilt angles of 7° and 45°. The measured data were evaluated with the RBX spectrum simulation code^19^. The effective thickness of the deposited layers was estimated assuming an atomic layer density of silicon dioxide, N = 6.9×10^22^ atom cm^−3^. Note in general RBS allows considerably accurate measurements of concentrations of constituents and the stoichiometry of thin films as a function of depth^20^. Recently, an assertion of 1% absolute accuracy (corresponding to 0.02–0.03 at. % accuracy in peak concentration) for RBS has been validated on the basis of the uncertainty budget^21^,^22^.

However, the sources of uncertainty in evaluation always depend on the properties of the material system to be analyzed and the given measurement conditions. In this work, considering the applied measurement statistics, background level, and the different components in the EDTS layers, we estimated an uncertainty of ±0.05 at. % for the evaluated Er contents.

The thickness and refractive index of the EDTS layer were characterized using a Metricon 2010 prism coupler and a 1320 nm laser was used to obtain the optical characteristics using the zero-order TE and TM modes. The EDTS samples were further evaluated using a Woollam M-2000DI rotating compensator spectroscopic ellipsometer for the refractive index (*n*) and extinction coefficient (*k*) at 1550 nm using multi-layer optical models. Standard excitation and emission photoluminescence (PL) spectra of the Er^3+^-ions in the bulk glasses and the EDTS samples were acquired using an Edinburgh Instruments FLS920 spectrometer. Furthermore, the steady state PL spectra of the bulk glass samples, under identical experimental setup, were obtained using an excitation wavelength of 980 nm, at a laser power of 1 mW. EDTS samples were characterized under the same setup, but excited using a higher laser power of 30 mW. The emission spectral range was scanned from 1400 to 1700 nm with 0.5 nm resolution. The PL lifetime was also evaluated using time resolved PL spectra, whereby the laser source was pulsed with a 100 ms period and a pulse width of 10 μs.

## Results and Discussion

**Figure 1:**
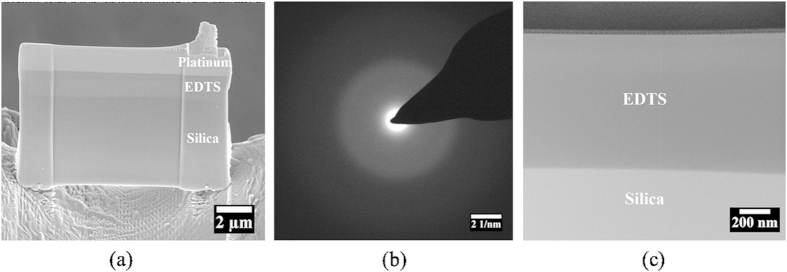
Distinct images of the EDTS layer on silica; (**a**) FIB-SEM image of the EDTS layer cross-section; (**b**) SAED pattern captured from the EDTS layer confirming the amorphous state; (**c**) HRTEM image of the interface between the EDTS layer and the silica substrate showing no clustering of Er^3+^-ions.

Representative micro-nano structural features of the EDTS were obtained through taking cross-sections of the sample surface by focused ion beam (FIB) lithography and subsequent imaging by HRTEM. Figure 1(a) presents a section removed by FIB, revealing a uniformly produced EDTS layer and the underlying pristine silica. A selected area electron diffraction (SAED) pattern, Fig. 1(b), captured from the centre of the modified layer resulted in a hollow ring. The appearance of such diffraction patterns indicate that no long range order exists after implantation, thus confirming the amorphous nature of the EDTS layer. An HRTEM cross-section is provided in Fig. 1(c), indicating a cluster-less homogeneous and well-defined boundary of the modified layer with the substrate.

### RBS analysis for elemental composition

**Figure 2:**
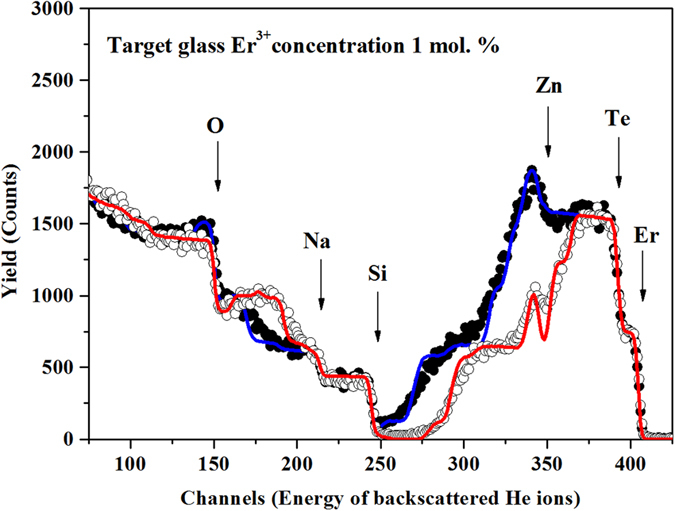
Typical RBS spectra of an EDTS layer on silica substrate measured at two different sample tilt angles of 7° (open dots) and 45° (solid dots), respectively. The corresponding RBX simulations are also shown (red and blue lines). Surface spectrum edges for Er, Te, Zn, Si, Na, and O are indicated.

The RBS analysis was carried out to quantify the elemental composition of the EDTS layer. Typical RBS spectra of the EDTS layer and the atomic composition for O, Si, Na, Zn, Te and Er in the EDTS layer are represented in Fig. 2 and Table 1, respectively.

From Table 1 it is evident that the EDTS layer contains approximately 60 at. % of O and the remainder is contributed by Si and the constituents of tellurite target glass. Importantly, the analysis confirms a record level of Er^3+^-ion concentrations quantified at 1.4 at. %, equivalent to 0.91 × 10^21^ atoms/cm^3^, which is the highest ever reported for a pure silica platform^7^,^8^. Our analysis shows that the doping concentration of Er^3+^-ions and of the matrix components remains essentially constant throughout the EDTS layer and there exists a transition layer that consists of very low concentrations (<10 at. %) of target glass components where the EDTS layer gradually transitions to the pristine silica region.

The amount of Te in EDTS layer is substantially low; despite its prevalence in the target glass. This may be due to the fact that during the fs-laser ablation, most of the volatile elements are likely to be lost during the flight from target to the substrate and affect the stoichiometry of the film^23^. Similarly a significant amount of Te, most volatile and structurally unstable component in the target glass, could deplete in the absence of proper oxygenation during the transport and results in the reduced concentrations of Te in EDTS.

**Figure 3:**
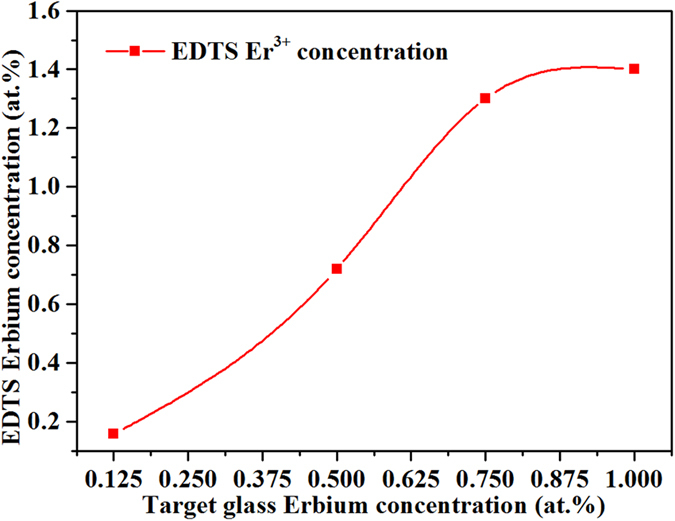
Erbium concentration in the EDTS layer vs. the corresponding erbium concentration in the initial tellurite target glass.

It is also observed that the effective EDTS layer thickness decreases with increasing Er^3+^-ion concentrations in the target glass. Figure 3 illustrate the relationship between the concentration of Er^3+^-ions in the target glass and the EDTS layer. This follows a nearly linear relationship at lower Er^3+^-ion concentrations and appears to saturate towards the higher concentration levels, in the given set of experimental data.

### EDTS planar optical layer characterization

**Figure 4:**
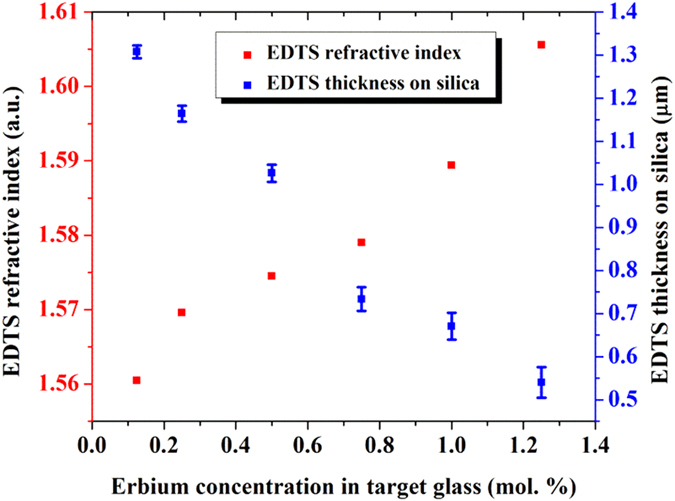
Thickness and refractive index trends for the EDTS layers with the Er^3+^-ion concentrations in the target glass.

The general trend of the EDTS layer thickness and refractive index with respect to the Er^3+^-ion concentrations in the target glasses, obtained through prism coupling is shown in Fig. 4. It is interesting to see that the thickness of the EDTS layer decreases, in agreement with RBS measurements, with increasing Er^3+^-ion concentrations in the target, while the refractive index follows an opposite trend. The refractive indices of the prepared samples were found in the range of 1.56 to 1.60 and the thickness of the optical layer reduces from 1.3 to 0.54 μm with increasing Er^3+^-ion concentrations.

The linear variation of the EDTS layer thickness and refractive index, with respect to Er^3+^-ion content, is representative of the density of the target material used. The replacement of tellurium with heavier Er^3+^-ions in the target glass gives rise to the denser packing of the material^24^. This was confirmed by measuring the density of the target glasses using Accupyc 1330 Pycnometer that showed an increase in density from 5.2078 to 5.2507 g/cm^3^ with the increased content of Er^3+^-ions from 0.125 to 1.25 mol%. The ablation rate for the dense layers will be less under constant laser energy, resulting in a lower transfer rate of the ablated material^25^ to the silica substrate, possibly decreasing the interfacial reaction and the rate of formation of EDTS leading to the reduced thickness. The increased Er^3+^-ion content, dense target material, doping in the silica glass network is intended to raise the density of the modified silica layer. This in turn results in the higher refractive index of EDTS layer with improved Er^3+^-ion concentrations. Similarly, the highly polarized trivalent Er^3+^-ions will generate more non-bridging oxygen in the glass network. This also aids the increase of refractive index, which is proportional to the polarizability, as non-bridging oxygen is significantly more polarized than bridging oxygen^26^.

The average *n* and *k* values obtained from the ellipsometry measurements corresponding to the higher index regions are summarized in Table 2. The refractive index shows a correlation with the Er^3+^-ion content as obtained previously by the prism coupler. The extinction coefficient also increases with Er^3+^-ion concentrations until 0.75 mol% and then decreased for 1 mol%.

The extinction co-efficient, which determines the overall material absorption, is a dipole sensitive property determined by the dopant ion-host interaction. In this respect the overall structural packing environment of the dopant may change depending upon the concentration of the Er^3+^-ion dopant, for example in EDTS. The reason for the extinction coefficient decreasing after a critical concentration can be that the dopant ion-host interaction may be decreasing with increasing Er^3+^-ion concentrations. Furthermore, because the minimum coordination of Er^3+^-ion is 6 for an octahedron case suggests that a larger volume might be necessary in a silica host to accommodate such ions. These structural changes need to be investigated further in detail.

### PL spectra

**Figure 5:**
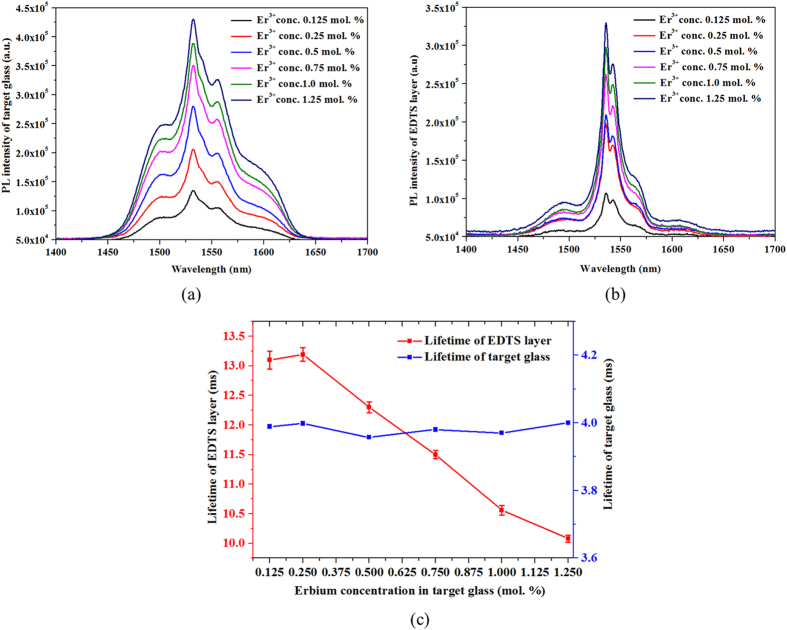
Photoluminescence spectra of the ^4^I_13/2_–^4^I_15/2_ transition of Er^3+^-ions with varying doping concentration in (a) tellurite target glass and (b) EDTS layer on silica. (**c**) Measured lifetime of EDTS and erbium-doped tellurite target glass as a function of Er^3+^-ion concentrations.

PL emission spectra for both bulk tellurite glasses and EDTS samples as a function of target glass Er^3+^-ion content are represented in Fig. 5(a,b), respectively.

A classic PL spectrum peaking at 1532 nm in tellurite target glass and 1535 nm in EDTS samples, corresponding to the observed ^4^I_13/2_–^4^I_15/2_ transition in Er^3+^-ions. Even though the Er^3+^-ions emission characteristics depend somewhat on the host glass material, the PL intensity increases with increasing Er^3+^-ion concentrations, both in the tellurite target glass as well in the EDTS layer. It is evident from the PL spectra that the broadband spectrum of tellurite glass target is modified to a narrower spectrum in the EDTS and this behaviour is similar to the reported emission spectra of silicates^27^. The measured typical full-width at half-maximum (FWHM) of the intensity profiles are 70 nm and 20 nm for the target glass and EDTS, respectively, and comparable to that reported^28^.

The variation of PL lifetime with respect to the Er^3+^-ions concentration in the target glass is shown in Fig. 5(c). The lifetime for the EDTS layer reduces with increasing Er^3+^-ion concentrations. According to Fig. 5(c), a 0.125 mol% Er^3+^-ion concentrations has a lifetime of 13.2 ms while that of 1.25 mol% is around 10 ms. The marginal lifetime decrease is due to the increase in the concentration of Er^3+^-ions which reduces the average spacing between the erbium ions^29^. Consequently, the electric-dipole interactions become more pronounced, facilitating energy transfer between Er^3+^-ions which contributes to the reduction in fluorescence lifetime.

It is remarkable that EDTS can contain such high concentrations of Er^3+^-ions while achieving the best ever lifetime of 10.56 ms, reported so far^8^. The multi-ion doping in silica helps to have a large distance between the active Er^3+^-ions, reducing the concentration quenching and enabling longer lifetimes. Recently adopted lifetime-density product^8^ for ascertaining the figure-of-merit for erbium doped materials is the highest, 0.96 × 10^19^ s cm^−3^, for this new glassy system. Such very high lifetimes validate the non-segregation of Er^3+^-ions in EDTS layer and shall contribute to strong PL emission and high optical gains.

## Conclusion

We have demonstrated a novel technique for integrating Er^3+^-doped tellurite material into silica glass using a femtosecond laser generated plasma implantation process to form equilibrium metastable EDTS layer. A very high concentration of Er doping with simultaneous implantation of Te-ions formulate high index contrast (>10%) optical layers on silica. EDTS can contain significantly high Er^3+^-ion concentrations of 1.4 at. % and the highest lifetime-density product 0.96 × 10^19^ s cm^−3^, thus offering the possibility to engineer large gain per unit length waveguide amplifiers. Six different tellurite target glasses with varying Er^3+^-ion concentrations were prepared to investigate the effect of target glass compositions in the formation of EDTS layer, highly beneficial for the fabrication of optical devices using this technique. The planar optical layer thickness, refractive index, PL intensity and lifetime can be precisely engineered by altering the Er^3+^-ion concentrations in the target glass. The reduction in EDTS layer thickness with increased Er^3+^-ion concentrations poses a challenge for the rate of fabrication which would need to be addressed for the commercial development of erbium doped integrated optic devices on silica using the ultrafast laser plasma approach reported here. Significantly, the cluster-free high refractive index/contrast^30^ EDTS, will facilitate the development of next generation SiPs with loss-compensated optical devices and amplifiers.

## Additional Information

**How to cite this article**: Chandrappan, J. *et al.* Target dependent femtosecond laser plasma implantation dynamics in enabling silica for high density erbium doping. *Sci. Rep.* **5**, 14037; doi: 10.1038/srep14037 (2015).

## Figures and Tables

**Table 1:** Parameters used in the RBX simulation of the measured RBS spectra.

Target glass composition (mol%)	EDTS layer composition (at. %)							
	Si	O	Er	Te	Zn	Na	EDTS layer eff. thickness (nm)	Transition layer eff. thickness (nm)
79.875TeO_2_-10ZnO-10Na_2_O-0.125Er_2_O_3_	23	57.2	0.157	2.8	6.3	10.5	1250	360
79.5TeO_2_-10ZnO-10Na_2_O-0.5Er_2_O_3_	17	60.5	0.72	2.9	4.9	14	1000	260
79.25TeO_2_-10ZnO-10Na_2_O-0.75Er_2_O_3_	18	58.6	1.3	2.6	7.5	12	520	230
79.0TeO_2_-10ZnO-10Na_2_O-1Er_2_O_3_	19	59.1	1.4	2.5	6	12	480	170

Effective thicknesses of the implanted layers given in nm are recalculated from thicknesses given in atom/cm^2^ (provided by the RBS analysis) assuming the atomic density of silica, respectively. The estimated uncertainty of the evaluated Er contents is ±0.05 at. %.

**Table 2:** EDTS layer refractive indices (*n*) and extinction coefficients (*k*) at the wavelength of 1550 nm.

Target glass composition (mol%)	EDTS layer	
	Refractive index	Extinction coefficient
79.875TeO_2_-10ZnO-10Na_2_O-0.125Er_2_O_3_	1.5754	0.0348
79.5TeO_2_-10ZnO-10Na_2_O-0.5Er_2_O_3_	1.5850	0.1051
79.25TeO_2_-10ZnO-10Na_2_O-0.75Er_2_O_3_	1.6438	0.1893
79.0TeO_2_-10ZnO-10Na_2_O-1Er_2_O_3_	1.6682	0.1491
